# Regional associations of white matter hyperintensities and early cortical amyloid pathology

**DOI:** 10.1093/braincomms/fcac150

**Published:** 2022-06-15

**Authors:** Luigi Lorenzini, Loes T Ansems, Isadora Lopes Alves, Silvia Ingala, David Vállez García, Jori Tomassen, Carole Sudre, Gemma Salvadó, Mahnaz Shekari, Gregory Operto, Anna Brugulat-Serrat, Gonzalo Sánchez-Benavides, Mara ten Kate, Betty Tijms, Alle Meije Wink, Henk J M M Mutsaerts, Anouk den Braber, Pieter Jelle Visser, Bart N M van Berckel, Juan Domingo Gispert, Frederik Barkhof, Lyduine E Collij, the EPAD consortium for the ALFA cohort, Annabella Beteta, Anna Brugulat, Raffaele Cacciaglia, Alba Cañas, Carme Deulofeu, Irene Cumplido, Ruth Dominguez, Maria Emilio, Karine Fauria, Sherezade Fuentes, Laura Hernandez, Gema Huesa, Jordi Huguet, Paula Marne, Tania Menchón, Albina Polo, Sandra Pradas, Blanca Rodriguez-Fernandez, Aleix Sala-Vila, Gonzalo Sánchez-Benavides, Anna Soteras, Marc Vilanova

**Affiliations:** Dept. of Radiology and Nuclear Medicine, Amsterdam University Medical Center, Amsterdam Neuroscience, Amsterdam, The Netherlands; Dept. of Radiology and Nuclear Medicine, Amsterdam University Medical Center, Amsterdam Neuroscience, Amsterdam, The Netherlands; Dept. of Radiology and Nuclear Medicine, Amsterdam University Medical Center, Amsterdam Neuroscience, Amsterdam, The Netherlands; Dept. of Radiology and Nuclear Medicine, Amsterdam University Medical Center, Amsterdam Neuroscience, Amsterdam, The Netherlands; Dept. of Radiology and Nuclear Medicine, Amsterdam University Medical Center, Amsterdam Neuroscience, Amsterdam, The Netherlands; Department of Neurology, Alzheimer Center Amsterdam, Amsterdam Neuroscience, Vrije Universiteit Amsterdam, Amsterdam UMC, Amsterdam, The Netherlands; Centre for Medical Image Computing (CMIC), Departments of Medical Physics & Biomedical Engineering and Computer Science, University College London, UK; MRC Unit for Lifelong Health and Ageing - University College London, UK; School of Biomedical Engineering, King’s College LondonUK; Barcelonaβeta Brain Research Center (BBRC), Pasqual Maragall Foundation, Barcelona, Spain; IMIM (Hospital del Mar Medical Research Institute), Barcelona, Spain; Barcelonaβeta Brain Research Center (BBRC), Pasqual Maragall Foundation, Barcelona, Spain; IMIM (Hospital del Mar Medical Research Institute), Barcelona, Spain; Universitat Pompeu Fabra, Barcelona, Spain; Barcelonaβeta Brain Research Center (BBRC), Pasqual Maragall Foundation, Barcelona, Spain; IMIM (Hospital del Mar Medical Research Institute), Barcelona, Spain; Centro de Investigación Biomédica en Red de Fragilidad Y Envejecimiento Saludable (CIBERFES), Madrid, Spain; Barcelonaβeta Brain Research Center (BBRC), Pasqual Maragall Foundation, Barcelona, Spain; IMIM (Hospital del Mar Medical Research Institute), Barcelona, Spain; Centro de Investigación Biomédica en Red de Fragilidad Y Envejecimiento Saludable (CIBERFES), Madrid, Spain; Atlantic Fellow for Equity in Brain Health at the University of California San Francisco, SanFrancisco, California, USA; Barcelonaβeta Brain Research Center (BBRC), Pasqual Maragall Foundation, Barcelona, Spain; IMIM (Hospital del Mar Medical Research Institute), Barcelona, Spain; Centro de Investigación Biomédica en Red de Fragilidad Y Envejecimiento Saludable (CIBERFES), Madrid, Spain; Dept. of Radiology and Nuclear Medicine, Amsterdam University Medical Center, Amsterdam Neuroscience, Amsterdam, The Netherlands; Department of Neurology, Alzheimer Center Amsterdam, Amsterdam Neuroscience, Vrije Universiteit Amsterdam, Amsterdam UMC, Amsterdam, The Netherlands; Department of Neurology, Alzheimer Center Amsterdam, Amsterdam Neuroscience, Vrije Universiteit Amsterdam, Amsterdam UMC, Amsterdam, The Netherlands; Dept. of Radiology and Nuclear Medicine, Amsterdam University Medical Center, Amsterdam Neuroscience, Amsterdam, The Netherlands; Dept. of Radiology and Nuclear Medicine, Amsterdam University Medical Center, Amsterdam Neuroscience, Amsterdam, The Netherlands; Department of Neurology, Alzheimer Center Amsterdam, Amsterdam Neuroscience, Vrije Universiteit Amsterdam, Amsterdam UMC, Amsterdam, The Netherlands; Department. of Biological Psychology, Vrije Universiteit Amsterdam, Neuroscience Amsterdam, Amsterdam, The Netherlands; Department of Neurology, Alzheimer Center Amsterdam, Amsterdam Neuroscience, Vrije Universiteit Amsterdam, Amsterdam UMC, Amsterdam, The Netherlands; Department of Psychiatry and Neuropsychology, School for Mental Health and Neuroscience, Maastricht University, Maastricht, The Netherlands; Dept. of Radiology and Nuclear Medicine, Amsterdam University Medical Center, Amsterdam Neuroscience, Amsterdam, The Netherlands; Barcelonaβeta Brain Research Center (BBRC), Pasqual Maragall Foundation, Barcelona, Spain; IMIM (Hospital del Mar Medical Research Institute), Barcelona, Spain; Universitat Pompeu Fabra, Barcelona, Spain; Centro de Investigación Biomédica en Red Bioingeniería, Biomateriales Y Nanomedicina, Madrid, Spain; Dept. of Radiology and Nuclear Medicine, Amsterdam University Medical Center, Amsterdam Neuroscience, Amsterdam, The Netherlands; Queen Square Institute of Neurology and Centre for Medical Image Computing, University College London, London, UK; Dept. of Radiology and Nuclear Medicine, Amsterdam University Medical Center, Amsterdam Neuroscience, Amsterdam, The Netherlands

**Keywords:** white matter hyperintensities, amyloid PET, regional associations, multivariate analysis, pre-dementia population

## Abstract

White matter hyperintensities (WMHs) have a heterogeneous aetiology, associated with both vascular risk factors and amyloidosis due to Alzheimer’s disease. While spatial distribution of both amyloid and WM lesions carry important information for the underlying pathogenic mechanisms, the regional relationship between these two pathologies and their joint contribution to early cognitive deterioration remains largely unexplored.

We included 662 non-demented participants from three Amyloid Imaging to Prevent Alzheimer’s disease (AMYPAD)-affiliated cohorts: EPAD-LCS (N = 176), ALFA+ (N = 310), and EMIF-AD PreclinAD Twin60++ (N = 176). Using PET imaging, cortical amyloid burden was assessed regionally within early accumulating regions (medial orbitofrontal, precuneus, and cuneus) and globally, using the Centiloid method. Regional WMH volume was computed using Bayesian Model Selection. Global associations between WMH, amyloid, and cardiovascular risk scores (Framingham and CAIDE) were assessed using linear models. Partial least square (PLS) regression was used to identify regional associations. Models were adjusted for age, sex, and *APOE-e4* status. Individual PLS scores were then related to cognitive performance in 4 domains (attention, memory, executive functioning, and language).

While no significant global association was found, the PLS model yielded two components of interest. In the first PLS component, a fronto-parietal WMH pattern was associated with medial orbitofrontal–precuneal amyloid, vascular risk, and age. Component 2 showed a posterior WMH pattern associated with precuneus-cuneus amyloid, less related to age or vascular risk. Component 1 was associated with lower performance in all cognitive domains, while component 2 only with worse memory.

In a large pre-dementia population, we observed two distinct patterns of regional associations between WMH and amyloid burden, and demonstrated their joint influence on cognitive processes. These two components could reflect the existence of vascular-dependent and -independent manifestations of WMH-amyloid regional association that might be related to distinct primary pathophysiology.

## Teaser

Lorenzini et al. describe two distinct regional patterns of association between white matter hyperintensities (WMHs) and early amyloid deposition, differentially related to vascular risk factors and cognitive performance. These results suggest the existence of vascular-dependent and -independent manifestations of WMH-amyloid regional association that might be related to distinct primary pathophysiology.

## Graphical Abstract

**Graphical abstract:**
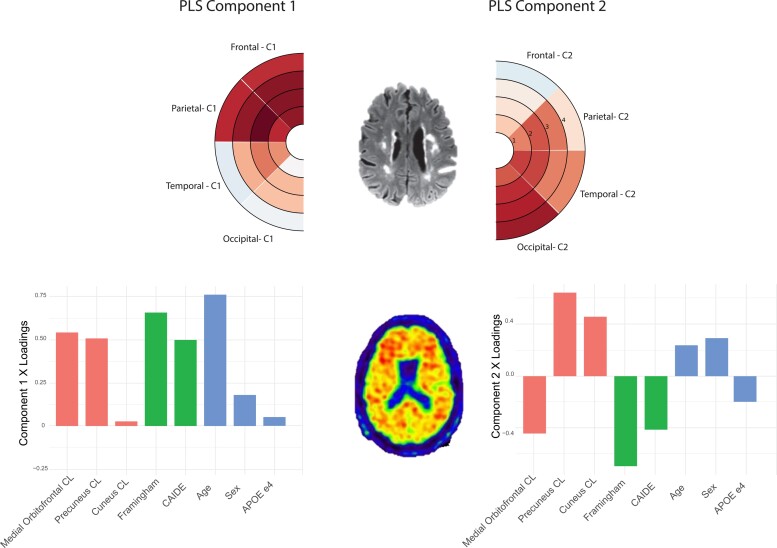

## Introduction

White matter hyperintensities (WMHs) and cortical amyloid deposition are imaging hallmarks of the two most common causes of dementia.^1^ WMHs, as measured on T_2_-weighted MRI, are markers of small-vessel disease (SVD). They are prevalent in cognitively unimpaired older individuals and associated with vascular risk factors.^2,3^ Amyloid-β pathology (Aβ) is hypothesized to initiate the Alzheimer’s disease pathological cascade,^4,5^ followed by the aggregation of intraneuronal hyperphosphorylated tau (p-tau), and eventual grey matter volume loss.^6,7^ Both WMH and amyloid burden can be observed years before the onset of cognitive symptoms and have been shown to contribute to cognitive impairment.^8,9^

Despite their diverse aetiologies, the frequent co-occurence of WMH and cerebral amyloid suggests a possible interaction of these two pathologies.^3^ For example, WMH-related impaired perivascular drainage has been shown to obstruct Aβ clearance, thus facilitating aggregation of Aβ proteins.^10^ In turn, cerebral amyloidosis can induce vasoconstriction and vessel wall damage, thus resulting in WMH development independently of vascular risk factors.^11^ Importantly, previous studies have underlined the spatial heterogeneity of WMH aetiology,^12^ showing that while systemic vascular factors relate to increased frontal WMH, posterior lesions are observed in relationship to amyloid deposition.^13,14^

However, to date the spatial distribution of amyloid burden has not been taken into account, while several early accumulating regions have been identified.^15^ Consequently, the correspondence and interaction of these two pathological image features has not been investigated on a regional level. Understanding the regional relationship between cerebral amyloid pathology and white matter damage, and its interplay with systemic vascular risk factors, is therefore important for an accurate characterization of the multifactorial processes leading to early cognitive impairment in neurodegenerative diseases.

In this work, we investigated the relationship between the regional distribution of WMH and amyloid burden in a large cohort of pre-dementia individuals from three parent cohorts involved in the Amyloid Imaging to Prevent Alzheimer’s disease (AMYPAD) consortium.^16^ To this aim, we first used linear analysis for the exploration of global association between WMH and amyloid. Then, we performed a multivariate partial least square (PLS) regression investigating the interplay between regional amyloid burden and systemic vascular risk factors in promoting differential patterns of WMH. Eventually, we assessed the joint contribution of these two pathologies to cognitive performance.

## Materials and methods

### Cohorts

Data were drawn from the ALFA+ cohort, European Medical Information Framework for Alzheimer’s disease—PreclinAD Twin60++ (hereafter PreclinAD), and European Prevention of Alzheimer’s Dementia Longitudinal Cohort Study (EPAD-LCS). All cohorts are affiliated with the AMYPAD Prognostic and Natural History Study (PNHS).^16^ The ALFA+ cohort is a nested longitudinal study of the ALFA (for Alzheimer’s and Families) study to characterize preclinical Alzheimer’s disease in cognitively unimpaired individuals who underwent amyloid PET imaging.^17^ The protocols have been approved by an independent Ethics Committee Parc de Salut Mar Barcelona and registered at Clinicaltrials.gov (ALFA+ Identifier: NCT02685969). The PreclinAD cohort included monozygotic twins from the Amsterdam sub-study of the European Medical Information Framework and is a longitudinal study on risk factors for amyloid pathology and cognitive decline in cognitively normal older adults.^18^ The study was approved by the VU University Medical Center’s ethics committee. The EPAD-LCS cohort recruited participants across 21 European sites across the full range of anticipated probability for Alzheimer’s disease development that aims at characterizing the preclinical and prodromal stages of Alzheimer’s disease, by creating a pool of well characterized individuals for recruitment in potential pharmacological trials.^19^ Institutional review boards of each participating centre approved the EPAD-LCS study.

### Participants

The ALFA+ cohort selected participants who were aged between 45 and 65 years enriched for family history of Alzheimer’s disease or *APOEε4* carriership and a global Clinical Dementia Rating (CDR) score of 0.^17,20^ The PreclinAD cohort inclusion criteria were having age above 60 years, a delayed recall score of >−1.5 SD of age-adjusted normative data on the Consortium to Establish a Registry for Alzheimer’s Disease (CERAD) 10-word list,^21^ a global CDR score of 0, Telephone Interview for Cognitive Status modified (TICS-m) score of 23 or higher,^22^ and a 15-item Geriatric Depression Scale score of <11.^23^ Eligibility criteria in the EPAD-LCS cohort were age above 50 years, CDR score of < 1, and no known diagnosis of dementia.^19^ For this study, we selected participants with PET and MRI <1 year apart from each other. Demographics and clinical characteristics were available from all cohorts. Individuals *APOE* genotyping was re-coded as being either ε4 non-carriers, ε4 heterozygous or ε4 homozygous. Owing to *APOE* ε2/ε4 genotype indeterminate relationship with Alzheimer’s disease and vascular risk factor, participants with this variant were excluded from the analysis. In total, 662 subjects were included in this study, of which 311 ALFA+, 176 PreclinAD and 175 EPAD-LCS subjects.

### Neuropsychological assessment

All participants underwent cognitive testing including the Mini-Mental State Examination (MMSE). Moreover, each cohort assessed cognitive domains using comprehensive standardized neuropsychological test batteries. Attention, memory (average between delayed and immediate memory in the EPAD cohort) and language cognitive domain scores were available across all cohorts. Therefore, we pulled cognitive performance on these three domains between cohorts by Z-scoring within each cognitive domain and cohort. Executive functioning domain was only available in PreclinAD and ALFA+ cohort. Visuo-constructional domain was only available in the EPAD cohort. Specific test and domain composite score description for each cohort can be found in the Supplementary Materials.

### Vascular risk scores

Individual vascular risk was computed by means of composite risk factor scores: the Cardiovascular Risk Factors, Aging, and Incidence of Dementia (CAIDE) and the Framingham Risk Score (FRS). The CAIDE score estimates the risk of developing late life dementia based on midlife vascular risk factors.^24^ The original scoring system for CAIDE uses information on age, sex, education, high blood pressure (BP), body mass index (BMI), total cholesterol and physical inactivity. The FRS is a sex-specific algorithm used to estimate the 10-year cardiovascular risk of an individual. It uses information on age, sex, systolic BP, antihypertensive medication, diabetes, total and HDL cholesterol and smoking.^25^ In the absence of blood biomarkers, self-reported hypercholesterolaemia was used to score cholesterol related information and re-coded as described in Calvin *et al*.^26^ Both scores were computed with and without including age, to check for possible over-corrections in later analysis.

### PET acquisition and processing

Amyloid PET scans from the ALFA+ (Siemens Biograph mCT scanner) and the PreclinAD (Philips Ingenuity Time-of-Flight PET–MRI scanner) cohorts were obtained with four frames (4 × 5 min) acquired 90–110 min post-injection of [^18^F]flutemetamol.^18,27^ Images were checked for motion and inter-frame registration was performed. Subsequently, the four frames from the PET images were averaged and registered to the corresponding 3D T_1_-weighted (T1w) MRI images. Then, the T1w images were registered to standard space and the same transformation was applied to the co-registered PET images, using SPM12.^18,27^ For the EPAD-LCS cohort, PET acquisition was performed under the AMYPAD PNHS protocol and consisted of four frames (4 × 5 min), acquired 90–110 min post-injection of [^18^F]flutemetamol or [^18^F]florbetaben.^16^ PET frames were averaged, co-registered to the corresponding MRI scans, and registered to the Montreal Neurological Institute space using SPM12.

PET images were intensity-normalized using the cerebellum as a reference region using the mask provided by the Centiloid (CL) project^28^ (http://www.gaain.org/centiloid-project). In the CL scale, CL = 0 corresponds with absence of amyloid in young controls, and CL = 100 corresponds with the burden of a typical mild-to-moderate Alzheimer’s disease dementia patient.^28^ Cortical Centiloid values were calculated using the standard target region and a previously calibrated conversion equation for ALFA+ and PreclinAD cohorts^29^ and the calibrated conversion equations for [^18^F]flutemetamol and [^18^F]florbetaben for the EPAD-LCS cohort.

Three regions of interest (ROIs) were created from the Learning Embeddings for Atlas Propagation (LEAP)^30^ atlas to capture amyloid pathology across early anterior and posterior regions:^31^ medial orbitofrontal cortex (gyrus rectus and medial frontal cortex), precuneus and cuneus. Regional standard uptake value ratios were extracted from these three ROIs and converted to regional Centiloid units using the global conversion equation.^28^

### MRI acquisition and processing

In the ALFA+ cohort, scans were obtained with a 3 T scanner (Ingenia CX, Philips Healthcare, Best, The Netherlands). The MRI protocol included a 3D T1w Turbo Field Echo (TFE) sequence (voxel size 0.75 × 0.75 × 0.75 mm, TR/TE: 9.90/4.6 ms, flip angle = 8°) and a 3D T2-FLAIR sequence (TSE, voxel size 1 × 1 × 1 mm, TR/TE/TI: 5000/312/1700 ms). Scans were visually assessed for quality and incidental findings by a trained neuroradiologist.^32^ In the PreclinAD cohort, MRI scans were obtained using a single 3 T scanner (Philips Ingenuity Time-of-Flight PET/MRI scanner) with a 8-channel head coil. The scan protocol included T1w sequences, acquired using sagittal turbo field echo sequence (1.00 mm^3^ isotropic voxels, TR/TE = 7.9 ms/4.5 ms, and flip angle = 8°), and 3D sagittal FLAIR sequences (1.12 mm^3^ isotropic voxels, TR/TE = 4800/279 ms, and inversion time = 1650 ms).^33^ The EPAD-LCS baseline data were acquired at 21 different EPAD-LCS sites. The core MRI protocol was performed on all consented participants and included 3D T1w, 3D FLAIR, 2D T2w and 2D T2* acquisition. Details on the MRI acquisition parameters and processing steps are previously described.^34^

WMH segmentation was performed using Bayesian Model Selection (BaMoS), an unsupervised model selection framework.^35^ Regional values of WMH were obtained by averaging lesions within atlas regions taking into account lobar boundaries (frontal, parietal, temporal, and occipital) and distance between the ventricular surface and cortex (4 layers) and depicted using bullseye plot representation.^36^ In this work, WMH regional values were averaged between the left and right hemisphere. Total intracranial volume was also calculated for normalization purposes, using a previously validated method.^37^

### Statistical analysis

All analyses were carried out in R version 4.0.3. Differences in demographics among the three cohorts were assessed using one-way ANOVA and Kruskal–Wallis tests for numerical and categorical variables, respectively. Data normalization for statistical analysis was performed within each cohort (Supplementary Materials).

#### Association between amyloid and global white matter hyperintensities burden

The cross-sectional association between global amyloid burden (independent variable) and normalized global WMH volumes (dependent variable) was assessed using linear models, with covariate adjustment by age, sex and *APOE* genotype. The same model was also run using each of the regional amyloid CL values and vascular risk scores as the main predictors.

#### Regional relationship between amyloid and white matter hyperintensities burden

Considering the high cross-correlations within the WMH and amyloid data sets, we assessed their regional relationship using PLS regression. PLS models relationships between two multivariate data sets, by finding linear combinations of predictors (e.g. regional amyloid) that maximally explain the variance in the outcome variables (e.g. regional WMH), maximizing the covariance between the two data sets.^38^ The contribution of each variable into each component is expressed as variable loadings, while PLS scores summarize individual observations, i.e. participants, into the estimated components.^39^

The PLS model was built in R using the *pls* package (version 2.7–3). Regional CL values, framingham and CAIDE vascular risk scores were included in the model as predictors. All the 16 regional WMH values were used as outcome measures. Age, sex, and *APOE* genotype were added to the predictors matrix as covariates. The best number of fitting latent components was determined using the cross-validation approach previously described.^38^

#### Relationship of partial least square components with cognitive domains

We further evaluated the joint contribution of regional WMH, regional amyloid, and vascular risk scores to cognitive performance. Linear models were used to investigate the effects of component scores onto performance in cognitive domains. Attention, memory and language performance were studied across cohorts, visuo-constructional performance in the EPAD participants only and executive functioning in the PreclinAD and ALFA+ only.

### Data Availability

The data used in this manuscript are available upon request from the specific projects involved.

## Results

### Cohort characteristics

Demographics and clinical characteristics are shown in Table 1. Mean age was 64.9 years (*SD* = 7.19), and 261 (39%) were male. Participants were without dementia (CDR = 0: *N* = 613, 92.60%; CDR = 0.5: *N* = 49, 7.40%) at inclusion with an average MMSE of 29.01 over the whole group. Higher global amyloid burden as expressed in CL units were observed in the EPAD participants compared with the other two cohorts. WMH load was lower in the ALFA+ cohort compared with EPAD and PreclinAD.

**Table 1:** Participants’ characteristics. Demographic and clinical characteristics of included participants are reported stratifying per cohort

	Whole population (*n* = 662)	ALFA (*n* = 311)	EPAD (*n* = 175)	PreclinAD (*n* = 176)
***Age (years)***	*64.90* (*7.19)*	*61.11* (*4.51)*	*66.29* (*6.98)*	*70.22* (*7.39)*
**Sex = M (*n*; %)**	261 (39.4)	113 (36.3)	77 (44.0)	71 (40.3)
***Education (years)***	*14.18* (*3.90)*	*13.43* (*3.53)*	*14.57* (*3.74)*	*15.11* (*4.43)*
***MMSE***	*29.01* (*1.23)*	*29.21* (*0.95)*	*28.73* (*1.63)*	*28.95* (*1.16)*
***CDR: 0.5 (%)***	*49* (*7.40)*	*0* (*0)*	*49* (*28)*	*0* (*0)*
***E4 carrier (n, %)***				
Non-carrier	369 (55.7)	143 (46.0)	105 (60.0)	121 (68.8)
Heterozygote ε4	242 (36.6)	136 (43.7)	57 (32.6)	49 (27.8)
Homozygote ε4	51 (7.7)	32 (10.3)	13 (7.4)	6 (3.4)
***Framingham Score***	*14.10* (*4.01)*	*11.76* (*2.83)*	*14.55* (*4.00)*	*16.87* (*3.46)*
***CAIDE score***	*6.78* (*2.08)*	*5.41* (*1.48)*	*7.54* (*1.91)*	*8.52* (*1.36)*
***Global Centiloid***	*12.32* (*26.60)*	*2.73* (*16.97)*	*26.92* (*35.08)*	*14.76* (*23.57)*
***WMH total volume (mL)***	*4543.90* (*6107.07)*	*3193.11* (*3726.43)*	*4798.18* (*5930.91)*	*6677.96* (*8568.13)*

MMSE = Mini-Mental State Examination; CDR = Clinical Dementia Rating score; CAIDE = Cardiovascular risk factors, aging and dementia; WMHs = White Matter Hyperintensities. Continuous variables are presented as mean (standard deviation), categorical variables as *n* (%). *P* < *0.001*

### Global white matter hyperintensities and cortical amyloid

Both global and regional amyloid burden did not show any significant effect on global values of WMH lesions. Vascular risk scores had a significant positive relationship with global WMH load, with high scores being related to greater WM lesions. Covariates coefficients showed significant influences of age on global WMH load, with older age being associated with more WM lesions (Table 2).

**Table 2:** Association of global and regional amyloid, vascular risk scores and covariates with global WMH volume

	Predictor	Estimate	Std. Error	*P*-value
Amyloid				
	Global CL	-0.009	0.014	0.51
	Precuneus CL	-0.001	0.012	0.91
	Medio-frontal CL	-0.021	0.013	0.11
	Cuneus CL	0.001	0.023	0.98
Vascular Risk Scores				
	Framingham Score	0.246	0.042	<0.001***
	CAIDE Score	0.098	0.039	<0.05*
Covariates				
	Age	0.384	0.039	<0.001 ***
	Sex	-0.001	0.073	0.98
	APOE ε4-Heterozygous	-0.076	0.079	0.33
	APOE ε4-Homozygous	0.269	0.142	0.058

Results from linear models investigating the effect of candidate variables on global WMH. *** denotes a *P*-value < 0.001; * denotes a *P*-value < 0.05. *CL = Centiloid; CAIDE = Cardiovascular risk factors, aging and dementia*.

### Multivariate white matter hyperintensities and cortical amyloid relationship

Cross-validation identified two components as being the optimal number of components, i.e. yielding the lowest root mean square error in the PLS model explaining regional variability in WMH (see Supplementary Materials).

**Figure 1:**
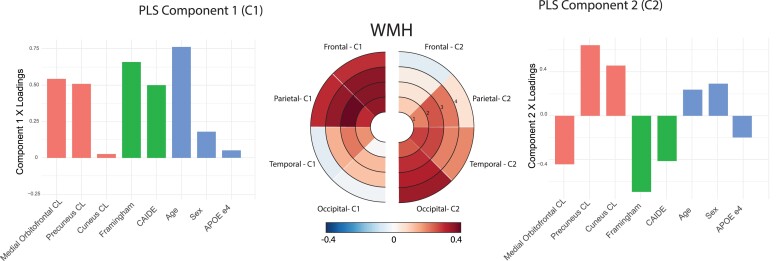
Association of amyloid and vascular risk scores with regional WMHs volumes. Results of the PLS regression analysis on 662 participants. *Left:* Loadings of the used variables into the first PLS component (C1). *Right:* Loadings of the used variables into the second PLS component (C2). WMH loadings (y loadings) are reported using bullseye plots representing lobes and layers as described in a study by Sudre *et al*.^36^ Loadings of regional amyloid (red, bar 1-3), vascular risk scores (green, bar 4-5) and covariates (blue, bar 6-8) are reported in a barplot (x loadings).

The first component (C1; Fig. 1) showed positive PLS loadings of frontal and parietal WMH, with highest values in the second and third, i.e. deep white matter layers. Temporal and occipital volumes of WMH demonstrated low contributions to this component. The contribution of the predictor variables, i.e. x loadings, showed that vascular risk scores had the highest PLS positive loadings to the C1 together with age. Medial orbitofrontal and precuneus amyloid measures were also positively related to this component, while cuneal amyloid showed no relationship.

In the second component (C2; Fig. 1), a posterior WMH regional pattern was found. A strong involvement of occipital WMH was observed for all layers with high positive PLS component loadings. Temporal and parietal WMH also showed mild positive contribution to C2 mostly in periventricular regions. Low PLS loadings were found for frontal WMH regions, with negative values in the 4th layer. This posterior pattern of WMH was mainly positively associated with precuneal and cuneal amyloid burden. Moreover, Framingham and CAIDE risk scores were negatively associated with C2. Compared to C1, this component was less related to age. All PLS loadings are reported in Supplementary Table 1 and Supplementary Table 2.

These results were highly consistent when correcting for cohort (Supplementary Figs 3–6).

### Relationship of partial least square components with cognitive domains

**Figure 2:**
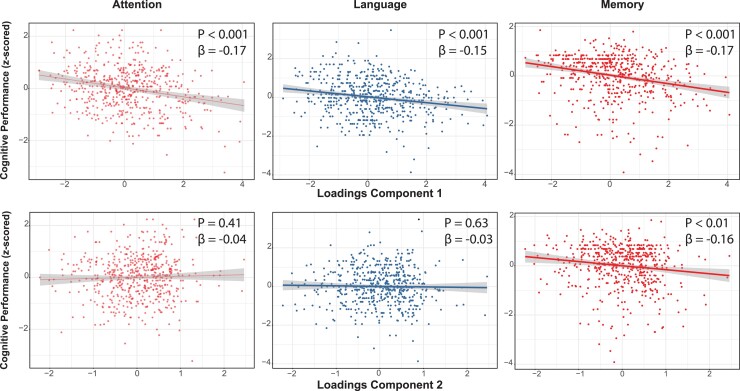
Association of PLS regression components with cognitive performance. Upper row: Component 1 (C1) scores’ relationship with attention (left), language (middle) and memory (right) performance across cohorts. Bottom row: Component 2 (C2) scores’ relationship with attention (left), language (middle) and memory (right) performance across cohorts. *P*-values (P) and beta coefficients (β) are reported.

We further used individual participants’ scores from the PLS model, i.e. the contribution of each observation to the components, to investigate their relationship with cognitive performance. Higher scores in the C1 were related to worse performance in memory, attention and language across cohorts, and with the executive functioning domain in the ALFA+ and PreclinAD. C1 was not related to visuo-constructional domain (only investigated in the EPAD cohort). By contrast, C2 scores only showed a significant negative association with the memory domain across cohorts (Fig. 2). Within-cohorts association is reported in Supplementary Fig. 7.

## Discussion

In this large multi-cohort study of pre-dementia participants, we found two spatial patterns of WMH associated with distinct profiles of regional cortical amyloid burden and vascular risk factors. First, fronto-parietal WMH load was related to amyloid burden in medial orbitofrontal and precuneus regions, with older age, and vascular risk factors. This component was related to cognitive functioning across all domains. Second, posterior WMH, mainly in occipital regions, was only positively associated with precuneus-cuneus amyloid. Higher scores of this component were related to worse memory functioning. These results suggest that the anterior and posterior WMH distributions are associated with different pathophysiologies, and advocate for a synergic contribution in promoting cognitive alterations typical of initial phases of neurodegenerative diseases.

Previous studies investigating the association of global WMH volume and amyloid burden have reported conflicting results.^40^ While there is some evidence of a positive relation across the Alzheimer’s disease spectrum,^41^ other studies and the current one identified no significant global association between these two pathologies on a whole brain level.^42^ However, the limited sensitivity of global scores to focal changes might have masked any regional associations between these two common pathologies. Using a multivariate statistical methodology, we provide further evidence for the existence of two interlinked regional patterns of WM lesions and amyloid deposition, highlighting the value of regional information for the understanding of the early interplay between these two pathologies.

In the first component, fronto-parietal lesions in the WM were positively related to medial orbitofrontal and precuneus amyloid burden, but also to vascular risk and age. In line with these results, recent neuropathological studies have observed frontal WMH in relationship to both SVD and amyloid-related degeneration.^13^ Neuroimaging findings have also reported a more vascular involvement in the pathogenesis of frontal WM lesions,^12^ putatively due the vulnerability of anterior and middle cerebral arteries to the development of SVD.^43^ Moreover, WMH lesions at baseline have been associated specifically to amyloid accumulation over time in frontal and parietal regions.^44^ This vascular-related WMH and amyloid pattern in the fronto-parietal lobes is consistent with a line of recent research suggesting a strong contribution of cerebrovascular dysfunction in the development of dementia due to Alzheimer’s disease.^45^ Specifically, the *two-hits* framework proposes cerebrovascular damage to be an early insult sufficient to initiate neuronal injury and neurodegeneration by itself, but that can also influence the amyloidogenic pathway to diminish Aβ clearance and increase Aβ production, leading to elevated amyloid levels in the brain.^46^ Considering the positive association of this component with older age, the proposed mechanism might be typical of the late-onset Alzheimer’s disease (LOAD) symptomatology.^47^ In fact, previous works have shown that vascular dysregulation would indeed be an early event in the pathogenesis of LOAD, further confirming the possible role of cerebrovascular dysfunction in initiating this pathological cascade.^48^ This component might therefore fournish neuroimaging evidence of the interplay between vascular and amyloid-related processes in the development of Alzheimer’s disease and stress the importance of considering individualized vasculo-protective approaches for early treatment. However, the lack of longitudinal imaging data does not allow for a causal interpretation of this process. Further studies are therefore needed to exemplify the temporal relationship between vascular factors and anterior WMH and amyloid.

The second component instead showed posterior WMH volumes being associated with cuneus and precuneus cortical amyloid, and negatively related to vascular factors. This result is in line with previous neuroimaging studies which observed a relationship of posterior WMH with amyloid positivity.^12,49^ In accordance with these findings, post-mortem studies have also found parietal WMH to be exclusively connected to AD-related Wallerian neurodegeneration and brain amyloid deposition, and unrelated to SVD.^14^ Our second component could therefore be consistent with a more pure Alzheimer’s disease pathway, where amyloid deposition acts as the primary mechanism promoting cortical neurodegeneration, subsequently causing WM demyelination and axonal loss.^50,51^ This result supports the idea of posterior WMH being a vascular-independent marker of early Alzheimer’s disease pathology and cerebral amyloidosis. The relationship of this component with younger age further indicates that this pattern could be typical of early-onset Alzheimer’s disease (EOAD). In line with this idea, EOAD has been observed to have a lower prevalence of vascular risk factors^52^ compared with the late-onset variant and to show increased parietal^53^ and occipital^54^ amyloid burden.

Considering the possible distinct aetiological underlying processes, it is of interest to investigate the two components relationship with cognitive functioning across domains. The first component showed a significant negative association with cognitive performance across domains, suggesting that the observed alteration associated with C1 could be mostly driven by the effect of aging.^55^ Nonetheless, the associations with memory and attention were stronger compared with visuo-construction and language performance. These cognitive domains are known to be affected by Alzheimer’s disease pathology and vascular burden, respectively.^9,56^ In contrast, the second component was only related to memory performance. This result is in line with previous findings of memory impairments in the early stages of the disease, and in relationship to primary amyloid pathology and posterior WMH.^9,57^ Nonetheless, the lack of association with other domains is in contrast to previous studies, which have shown that early manifestations of Alzheimer’s disease are usually characterized by a worse cognitive impairment and a greater decline of cognitive functions over time.^58^ This discrepancy could be due to the fact that we only included non-demented subjects and thus did not have the appropiate population to observe strong assocations between C2 and cognitive functioning. Indeed, when stratifying the analysis based on age (<70), we observed stronger association of C2 with memory performance (β = -0.21; *P* < 0.01; Supplementary Fig. 8).

This study lacks longitudinal data, hampering the interpretation of directionality and causality of the observed effects. Moreover, unavailability of longitudinal cognitive data could hinder the identification of a significant relationship between C2 and non-amnestic cognitive performance. Future longitudinal studies are encouraged to confirm the present results, for predicting distinct cognitive trajectories. Second, in this work we hypothesized possible differential aetiological causes underlying the two spatial components. However, previous work has suggested that other types of vascular pathology are also distinctly related to either Alzheimer’s disease pathology or vascular risk factors. For example, occipital amyloid burden has been linked to cerebral amyloid angiopathy (CAA), a form of SVD which predominantly occurs in posterior areas of the brain and is the direct result of Aβ deposition in the vessel walls.^59^ In addition, the presence of (posterior) CAA has been associated with higher prevalence of *APOE*-ε4 carriership and posterior and specifically occipital WMH burden,^60^ providing further support for a vascular-independent pathway of WMH pathology. Future workould therefore investigate multiple aspects of vascular dysfunction to shed light on the underlying mechanistic interplay between WMH and amyloid pathology.

## Conclusion

Our multivariate approach provided evidence for two patterns of WMH lesions and amyloid burden; a fronto-parietal co-occurrence mainly related to age and vascular risk factors, and a posterior association independent of vascular determinants. These two components could reflect the existence of vascular-dependent and -independent manifestations of WMH-amyloid regional association that might be related to distinct primary pathophysiology. Understanding this pathophysiological interplay could be of value in uncovering the pathogenesis of cognitive impairment.

## Supplementary Material

fcac150_Supplementary_Data

## Appendix

Collaborators of the ALFA study are: Annabella Beteta, Anna Brugulat, Raffaele Cacciaglia, Alba Cañas, Carme Deulofeu, Irene Cumplido, Ruth Dominguez, Maria Emilio, Karine Fauria, Sherezade Fuentes, Laura Hernandez, Gema Huesa, Jordi Huguet, Paula Marne, Tania Menchón, Albina Polo, Sandra Pradas, Blanca Rodriguez-Fernandez, Aleix Sala-Vila, Gonzalo Sánchez-Benavides, Anna Soteras and Marc Vilanova.

## Abbreviations

ALFA =: Alzheimer and Families
AMYPAD =: Amyloid Imaging to Prevent Alzheimer’s disease
CAIDE =: Cardiovascular Risk Factors, Aging, and Incidence of Dementia
CDR =: Clinical Dementia Rating
CL =: Centiloid
EOAD =: early-onset Alzheimer’s disease
EPAD-LCS =: European Prevention of Alzheimer’s Dementia Longitudinal Cohort Study
LOAD =: late-onset Alzheimer’s disease
MMSE =: Mini-Mental State Examination
PLS =: partial least square
ROI =: region of interest
SVD =: small-vessel disease
WMHs =: white matter hyperintensities.
