# Supplementary material for: Regional associations of white matter hyperintensities and early cortical amyloid pathology
Source: Brain Commun. 2022 Jun 15;4(3):fcac150. doi: 10.1093/braincomms/fcac150 (PMC9246276; doi:10.1093/braincomms/fcac150)
Supplement: fcac150_Supplementary_Data [file fcac150_supplementary_data.docx]

Supplementary Materials

# Neuropsychological Assessment

As specified in the main text, only memory, attention and language cognitive domains were available for all cohorts and therefore pulled together. Each cohort assesed and defined cognitive domains independently. In the PreclinAD cohort, cognitive functioning was assessed with a standardized neuropsychological test battery covering four domains: memory, attention, executive functioning, and language [^1^](https://paperpile.com/c/71f6RM/9NgZl). For the memory domain, the total immediate recall and delayed recognition of the Dutch version of the Rey Auditory Verbal Learning Test (RAVLT), the three and twenty minute recall of the Rey Complex Figure Test (RCFT) and the total score of the FNAME-names and -occupation delayed recall were used. For the attention domain, the Trail Making Test (TMT) part A, the Digit Symbol Substitution Test and the forward condition of the Digit Span were used. For the executive function domain, the TMT part B corrected for TMT part A, the backward condition of the Digit Span, the Dutch version of the Controlled Oral Word Association Test (letter fluency), with letters D A T were used. For the language domain, the category fluency (animal fluency) one minute and the graded naming test (GNT) were used. In the EPAD cohort, the Repeatable Battery for the Assessment of Neuropsychological Status (RBANS; [^2^](https://paperpile.com/c/71f6RM/qVwSI)) was performed to evaluate five cognitive domains: attention, language, delayed memory, immediate memory and visuo-constructional indices [^2^](https://paperpile.com/c/71f6RM/qVwSI). In the ALFA+ cohort, we computed memory, attention, executive functioning and language composite domain scores. For the memory domain, the Free and Cued Selective Reminding Test total immediate free recall and total delayed record were used. For the attention domain, the TMT part A, the S-span test and the forward condition of the Digit Span were used. For the executive domain, we averaged scores at the TMT part B corrected for TMT part A test, the matrix test, the coding test and the Digit Span backward and sequencing condition. For the language domain, the semantic fluency test was used.

# Centiloid Normalization

Normalization of amyloid centiloid (CL) values included in the analysis was performed within each cohort. Following previous work [^3^](https://paperpile.com/c/71f6RM/TvrYo), we normalized CL by using Gaussian Mixture Modeling (GMM; mixtools and AdaptGauss packages from R statistical software program). GMM is a probabilistic model for representing normally distributed subpopulations within an overall population. Since amyloid deposition often follows a bimodal distribution, we used the mean and standard deviation of the first gaussian (i.e. amyloid negative group) distribution were then used to scale the data. Figure S1 shows the output of GMM fitting on cohort-specific global centiloid data. Regional values were distributed similarly to global, and are not shown here.


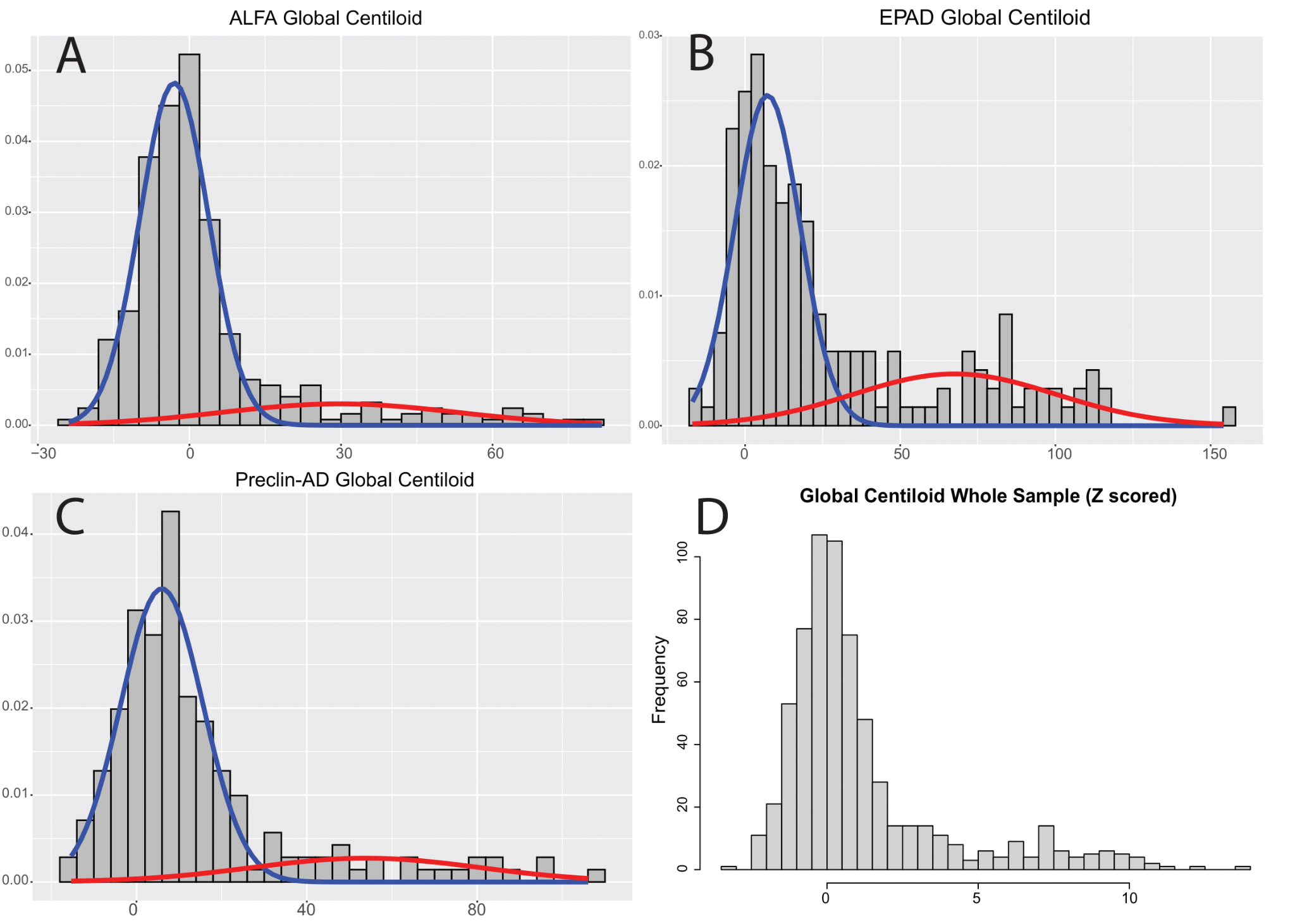


**Supplementary figure 1. Gaussian Mixture Modeling on cohort specific global centiloid data.** GMM fits two components into the distribution, finding a “normal” (in blue) and “pathological” (in red) population. A = ALFA global gentiloid; B = EPAD global centiloid; C = Preclin-AD global centiloid; D = distribution of global centiloid values on the whole cohort after normalization with GMM.

# White Matter Hyperintensities Automated Segmentation and Volumes Normalization

BaMoS uses a Gaussian mixture model to mutually model healthy tissue and unexpected observations. The number of required Gaussian components is dynamically optimized using the Bayesian Inference Criterion. Once the data model is fitted, the actual lesion segmentation is performed by voxel wise comparison to normal appearing white matter and only the supratentorial WMH volumes located in the white matter and subcortical gray matter are included [^4^](https://paperpile.com/c/71f6RM/0gdJe). All analysis used WMH expressed as a percentage of the TIV to account for total brain size.

In young and pre-clinical populations, white matter hyperintensities (WMH) often present a highly right-skewed distribution, due to the presence of individuals with no or minimal lesions. Such distributions prevent the use and interpretation of typical parametric statistics. Non-normality of global WMH volumes was confirmed in our cohort through the Shapiro-Wilk test with a p-value < 0.001. For this reason, we normalized WMH data using rank-based inverse normal transformation (RNOmni package from R statistical software program), replacing each observation by its fractional rank within the array [^5^](https://paperpile.com/c/71f6RM/nbess). **Figure S2** shows global WMH distribution before and after rank normalization. The same procedure was applied on regional values (not shown).


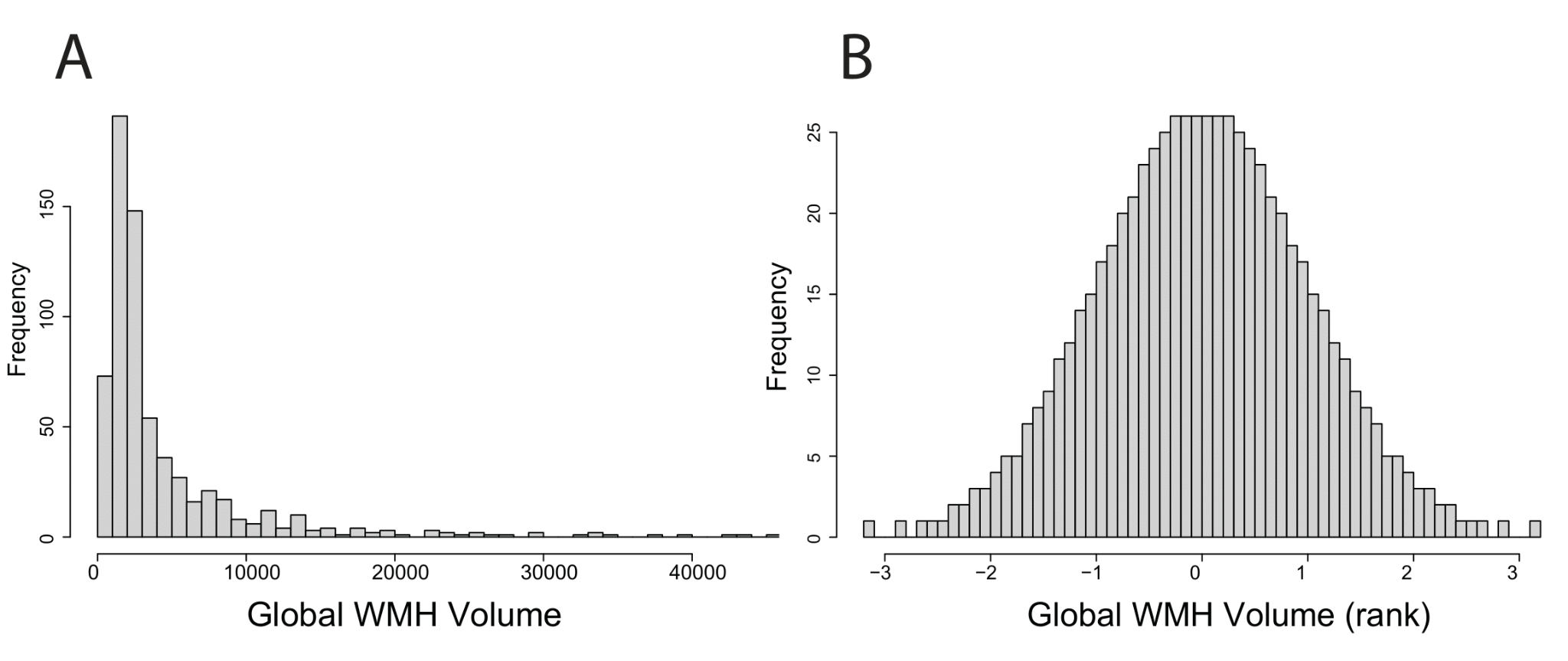


**Supplementary figure 2. Global white matter hyperintensities volumes normalization.** A) Right-skewed distribution of raw global WMH volumes; B) Normal distribution of ranked global WMH volumes.

# Selecting the number of component in PLS analysis

In PLS regression, the number of components to be used in the model is considered a parameter or tuning parameter. For this reason, the optimal number of K PLS components must be derived using resampling methods. In this work, we chose to use K-fold cross-validation as a resampling method for selection of the optimal number of components. Strong evidence exists in support of cross-validation in PLS regression [^6,7^](https://paperpile.com/c/71f6RM/RntC9+7PiMS).

To carry out cross-validation, the data were split into 10 randomly selected folds. The 10 segments were then used as test datasets in an iterative process, fitting the PLS regression model on the remaining data and testing it on the left out segment. For each iteration, PLS models with 1 to rank(X) number of components were fitted, with X being the number of variables in the predictors matrix. Cross-validation errors were stored across iterations. The number of components with the smallest cross-validation error across iteration was selected as being 2.

# PLS loadings

As described in the main text, PLS output can be summarized using loadings and scores. PLS loadings represent the contribution of each variable in the X and Y matrices to the latent factors (or components). In this paragraph, we report variable loadings of our response (Y) matrix (Table S1) predictor (X) matrix (Table S1).

**Supplementary table 1. PLS loadings of y matrix (WMH).** WMH volumes are stratified by lobes and layers (distance from the ventricles). For each region of interest, the loading of that variable to the PLS components 1 and 2 are reported.

|  | Component 1 | Component 2 |
| --- | --- | --- |
| Frontal 1 | 0.35 | 0.10 |
| Frontal 2 | 0.37 | 0.05 |
| Frontal 3 | 0.36 | 0.03 |
| Frontal 4 | 0.29 | -0.05 |
| Parietal 1 | 0.30 | 0.20 |
| Parietal 2 | 0.40 | 0.25 |
| Parietal 3 | 0.35 | 0.21 |
| Parietal 4 | 0.30 | 0.06 |
| Occipital 1 | 0.01 | 0.24 |
| Occipital 2 | 0.13 | 0.30 |
| Occipital 3 | 0.12 | 0.32 |
| Occipital 4 | -0.02 | 0.34 |
| Temporal 1 | 0.18 | 0.28 |
| Temporal 2 | 0.21 | 0.27 |
| Temporal 3 | 0.14 | 0.20 |
| Temporal 4 | -0.04 | 0.19 |

**Supplementary table 2. PLS loadings of X matrix (Amyloid, vascular risk scores and ).** For each early accumulating amyloid region of interest, the loading of that variable to the PLS components 1 and 2 are reported.

|  | Component 1 | Component 2 |
| --- | --- | --- |
| Medio-Frontal Amyloid | 0.53 | -0.45 |
| Precuneus Amyloid | 0.51 | 0.65 |
| Cuneus Amyloid | 0.02 | 0.56 |
| Framingham Score | 0.64 | -0.68 |
| CAIDE Score | 0.50 | -0.41 |
| Age (years) | 0.77 | 0.22 |
| Sex | 0.20 | 0.30 |
| E4 Carriers | 0.02 | -0.20 |

# Cohort Correction

Participants included in the study were retrieved from three different cohorts. While all cohorts specifically targeted preclinical stages of Alzheimer’s disease, some heterogeneity could still be observed in demographics and pathological characteristics (Table 1 in main text). For this reason, we evaluated differences in PLS scores between cohorts using an analysis of variance (ANOVA).

For the first component, Preclin-AD showed the highest scores (B = 1.75, P-value < 0.0001), followed by EPAD (B = 0.94, P<0.0001; Figure S3). For the second component, similar scores were found between the three cohorts, with only Preclin-AD showing the lower scores this time (B = -0.28, P-value < 0.001; Figure S4).


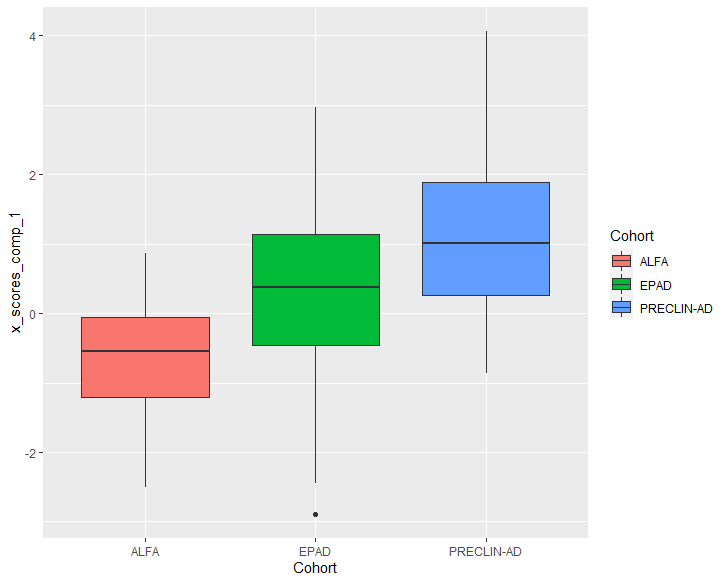


**Supplementary figure 3. Component 1 participant scores across the three cohorts**


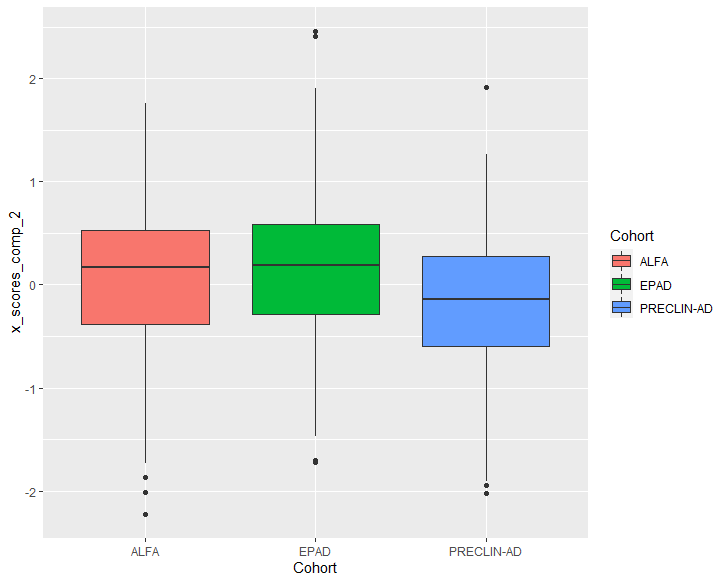


**Supplementary figure 4. Component 2 participant scores across the three cohorts.**

Further, we evaluated the effect of correcting for cohort in the main PLS model. To do so, we first computed the residuals of two models having the cohort as a predictor factor and the y (WMH) and x (amyloid) as an outcome, respectively. Those residuals were then used as inputs to the PLS model. The results of this correction were highly consistent with our main analysis and are reported in Figure S5 and S6.


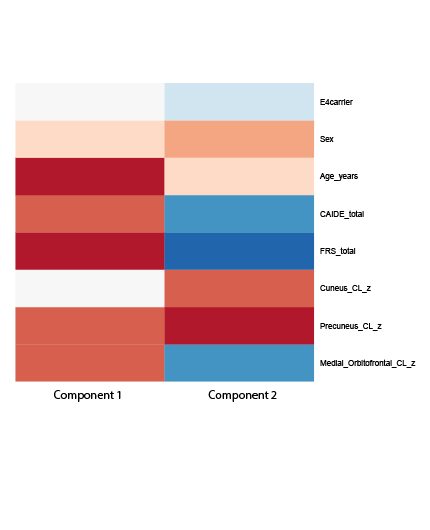


**Supplementary figure 5. Component X loadings after cohort correction.**


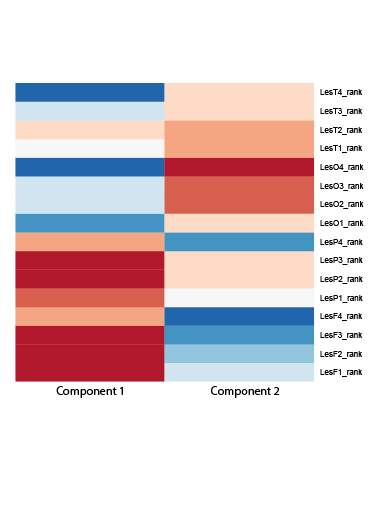


**Supplementary figure 6. Component Y loadings after cohort correction.**

# Relationship of PLS scores with Cognitive Performance

In the main text of this work, we only report association of PLS individual scores, i.e. contribution of each observation (participant) to the latent factors (components), across cohorts. This analysis allowed for a stronger statistical power due to bigger sample size. However, pulling cognitive data not only reduced the number of available cognitive domains ( only 3 in common between the two cohorts) but might also expose the analysis to biases due to inter-cohort normalization steps. For this reason, we further explored the effect of PLS scores on cognitive domains within each cohort separately. Results are consistent with the analysis reported in the main text (**Figure S3**).


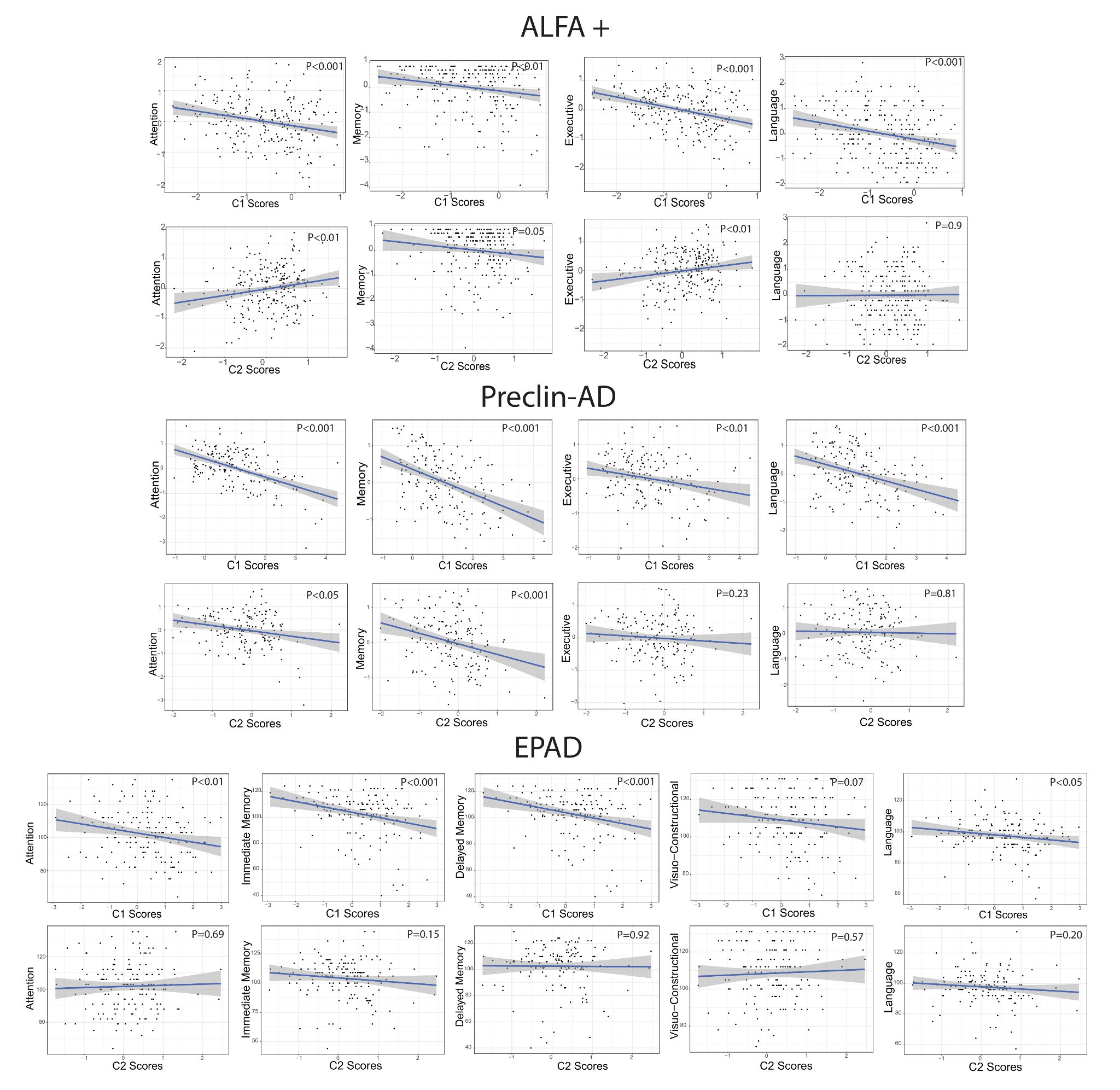


**Supplementary figure 7. Association of PLS scores with cognitive domains within cohorts.** Scatter plots visualizing the association between component 1 (C1) and 2 (C2) scores and cognitive domains, for the ALFA + (uppen panel), PreclinAD (middle panel) and EPAD (lower panel) cohort separately.

To further confirm the hypothesized relationship of component two with the early forms of AD, we run a confirmatory analysis evaluating the relationship of C2 scores with cognitive performance in the memory domain, in individuals with age below 70 years. Similar to our main analysis, we found that C2 scores significantly predicted memory performance and observed lower p-values (p < 0.01) and higher beta estimates (-0.21) (Figure S4).


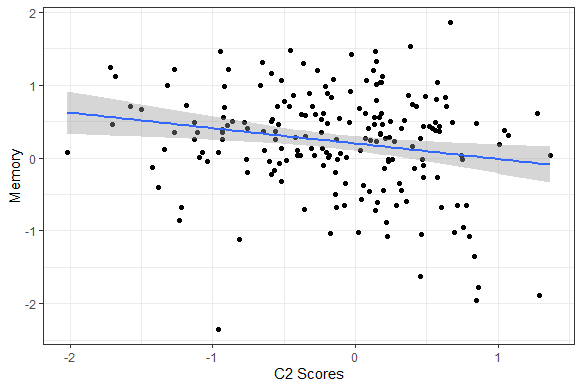


**Supplementary figure 8. Association of C2 individual scores with memory performance in participants with age < 70.**

1. [Konijnenberg E, den Braber A, Ten Kate M, et al. Association of amyloid pathology with memory performance and cognitive complaints in cognitively normal older adults: a monozygotic twin study. *Neurobiol Aging*. 2019;77:58-65.](http://paperpile.com/b/71f6RM/9NgZl)

2. [Randolph C, Tierney MC, Mohr E, Chase TN. The Repeatable Battery for the Assessment of Neuropsychological Status (RBANS): Preliminary Clinical Validity. *Journal of Clinical and Experimental Neuropsychology*. 1998;20(3):310-319. doi:](http://paperpile.com/b/71f6RM/qVwSI)[10.1076/jcen.20.3.310.823](http://dx.doi.org/10.1076/jcen.20.3.310.823)

3. [Collij LE, Heeman F, Salvadó G, et al. Multitracer model for staging cortical amyloid deposition using PET imaging. *Neurology*. 2020;95(11):e1538-e1553.](http://paperpile.com/b/71f6RM/TvrYo)

4. [Sudre CH, Cardoso MJ, Bouvy WH, Biessels GJ, Barnes J, Ourselin S. Bayesian model selection for pathological neuroimaging data applied to white matter lesion segmentation. *IEEE Trans Med Imaging*. 2015;34(10):2079-2102.](http://paperpile.com/b/71f6RM/0gdJe)

5. [McCaw ZR, Lane JM, Saxena R, Redline S, Lin X. Operating Characteristics of the Rank-Based Inverse Normal Transformation for Quantitative Trait Analysis in Genome-Wide Association Studies. doi:](http://paperpile.com/b/71f6RM/nbess)[10.1101/635706](http://dx.doi.org/10.1101/635706)

6. [Yoshida K, Shimizu Y, Yoshimoto J, et al. Prediction of clinical depression scores and detection of changes in whole-brain using resting-state functional MRI data with partial least squares regression. *PLoS One*. 2017;12(7):e0179638.](http://paperpile.com/b/71f6RM/RntC9)

7. [Höskuldsson A. Variable and subset selection in PLS regression. *Chemometrics Intellig Lab Syst*. 2001;55(1):23-38.](http://paperpile.com/b/71f6RM/7PiMS)
